# Glutamine Synthetase Contributes to the Regulation of Growth, Conidiation, Sclerotia Development, and Resistance to Oxidative Stress in the Fungus *Aspergillus flavus*

**DOI:** 10.3390/toxins14120822

**Published:** 2022-11-23

**Authors:** Sen Wang, Ranxun Lin, Elisabeth Tumukunde, Wanlin Zeng, Qian Bao, Shihua Wang, Yu Wang

**Affiliations:** State Key Laboratory of Ecological Pest Control for Fujian and Taiwan Crops, Key Laboratory of Pathogenic Fungi and Mycotoxins of Fujian Province, and School of Life Sciences, Fujian Agriculture and Forestry University, Fuzhou 350002, China

**Keywords:** *Aspergillus flavus* (*A. flavus*), glutamine synthetase, reactive oxygen species (ROS), L-α-aminoadipic acid

## Abstract

The basic biological function of glutamine synthetase (Gs) is to catalyze the conversion of ammonium and glutamate to glutamine. This synthetase also performs other biological functions. However, the roles of Gs in fungi, especially in filamentous fungi, are not fully understood. Here, we found that conditional disruption of glutamine synthetase (AflGsA) gene expression in *Aspergillus flavus* by using a xylose promoter leads to a complete glutamine deficiency. Supplementation of glutamine could restore the nutritional deficiency caused by AflGsA expression deficiency. Additionally, by using the xylose promoter for the downregulation of *AflgsA* expression, we found that AflGsA regulates spore and sclerotic development by regulating the transcriptional levels of sporulation genes *abaA* and *brlA* and the sclerotic generation genes *nsdC* and *nsdD*, respectively. In addition, AflGsA was found to maintain the balance of reactive oxygen species (ROS) and to aid in resisting oxidative stress. AflGsA is also involved in the regulation of light signals through the production of glutamine. The results also showed that the recombinant AflGsA had glutamine synthetase activity in vitro and required the assistance of metal ions. The inhibitor molecule L-α-aminoadipic acid suppressed the activity of rAflGsA in vitro and disrupted the morphogenesis of spores, sclerotia, and colonies in *A. flavus*. These results provide a mechanistic link between nutrition metabolism and glutamine synthetase in *A. flavus* and suggest a strategy for the prevention of fungal infection.

## 1. Introduction

*Aspergillus flavus* (*A. flavus*) is a saprophytic, pathogenic, and conditional plant fungus that invades important crops such as peanuts and corn during storage and transportation [1,2,3]. *A. flavus* is not only a plant pathogenic fungus but also causes invasive aspergillosis, threatening human life [4,5]. In addition, this fungus produces toxic secondary metabolites (aflatoxins) that have been recognized as notorious carcinogenic natural contaminants since their discovery [6,7,8]. Aflatoxin B1 (AFB1) has strong carcinogenic, teratogenic, and genotoxic properties [9]. Understanding the mechanisms of development and toxin synthesis of *A. flavus* can greatly improve the control strategies for fungal contamination. Therefore, how to effectively prevent and inhibit the infestation of *A. flavus* has become a major challenge.

Nitrogen metabolism is an important part of an organism’s metabolism. Glutamine synthetase (Gs) is one of the key central enzymes in the nitrogen metabolic pathway [10] that catalyzes the conversion of glutamate and ammonium to glutamine via an ATP-dependent reaction [11]. Although Gs performs a variety of divergent cellular functions such as nitrogen metabolism and amino acid production [12,13,14,15], its biological function is not limited to glutamine synthesis. In wheat (*Triticum aestivum* L.), Gs controls the nitrogen cycle during plant growth and development [16]. Surprisingly, suppression of the Gs gene causes impaired photosynthesis and photorespiration, leading to a significant accumulation of reactive oxygen species (ROS) [17] in *Amaranthus palmeri* (*A. palmeri*). The Gs gene promotes organism repair via cellular nucleotide synthesis after DNA damage [18] and maintains osmotic homeostasis [19]. In *Schizosaccharomyces pombe* (*S. pombe*) and *Aspergillus nidulans* (*A. nidulans*), the inactivation of Gs leads to growth and developmental retardation via glutamine-dependent malnutrition [20,21]. Gs loss of function affects the primary and secondary metabolites’ synthesis in the phytopathogenic fungus *Gibberella fujikuroi* (*G. fujikuroi*) [15].

In addition, glutamine synthetase has emerged as a new target for drug discovery and design. Methionine sulfoximine is used as a classical inhibitor for Gs protein activity in bacteria [11]. In plants, the role of Gs makes it an important target for the herbicide glufosinate [22]. Generally, Gs is classified into three types based on molecular weight and 3D spatial structure: GsI, GsII, and GsIII [23]. The Gs-encoding genes in filamentous fungi are usually identified as belonging to the GsII family [24]. Although the amino acid sequences are quite different for the three types of Gs enzymes, these proteins share similar tetrameric geometric structures consisting of two oligomeric rings in a duplex symmetry [25,26,27,28]. This oligomerization of Gs indicates that the protein may interact with other molecules and perform their functions in vivo. Indeed, a series of small molecules have been reported to decrease Gs activity, including amino acids, carbamoyl phosphate, and glucosamine-6-phosphate [11,29].

Here, we report that the AflGsA protein is important for colony growth, conidia production, and sclerotia development in *A. flavus*. Furthermore, AflGsA is involved in balancing ROS and resisting oxidative stress. L-α-aminoadipic acid, a potent inhibitor of rAflGsA, was effective in inhibiting growth, spore, and sclerotia production in *A. flavus*. These results provide detailed and comprehensive information concerning the regulatory mechanism of AflGsA in *A. flavus*.

## 2. Results

### 2.1. Identification of AflGsA in A. flavus

**Figure 1:**
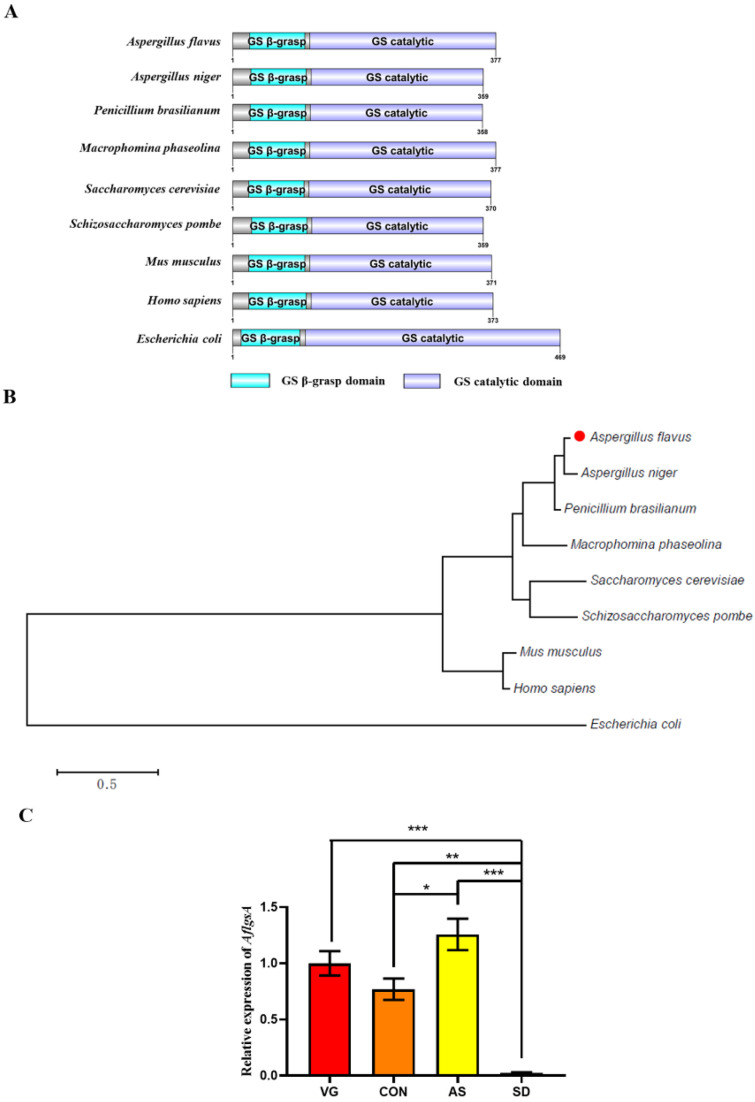
Sequence analysis and expression profiles of AflGsA in *A. flavus*. (**A**) Domain structure analysis of AflGsA from *A. flavus* and other species (*Aspergillus niger*, *Penicillium brasilianum*, *Macrophomina phaseolina*, *S. cerevisiae*, *S. pombe*, *Mus musculus*, *Homo sapiens*, and *Escherichia coli*). (**B**) Phylogenetic tree of AflGsA from different species. (**C**) Expression patterns of *AflgsA* were tested by qRT-PCR at vegetative growth (VG), conidial development (CON), aflatoxin synthesis (AS), and sclerotial development (SD) stages. * indicates a significance level of *p* < 0.05, ** indicates a significance level of *p* < 0.01, and *** indicates a significance level of *p* < 0.001 based on one-way ANOVA with three biological replicates.

The sequence of the *A. flavus* Gs (AflGsA) protein was obtained from the NCBI gene database (AFLA_051930), which exhibited 63% similarity to GsA in *Saccharomyces cerevisiae* (*S. cerevisiae*). All of the analyzed proteins contained two conserved domains (Figure 1A). The phylogenetic tree analysis based on AflGsA and other homologous proteins showed that AflGsA was highly conserved in *Aspergillus* spp. (Figure 1B). The expression profiles of *AflgsA* were monitored by quantitative real-time PCR (qRT-PCR) at vegetative growth (VG), conidial development (CON), aflatoxin synthesis (AS), and sclerotial development (SD) stages [30]. The transcript level of *AflgsA* was the highest in the AS stage, whereas the lowest transcript level was reached in the SD stage (Figure 1C). The differences in expression patterns suggest that *AflgsA* may perform different functions at different stages in *A. flavus*.

### 2.2. AflgsA Is an Essential Gene for the Growth of A. flavus

**Figure 2:**
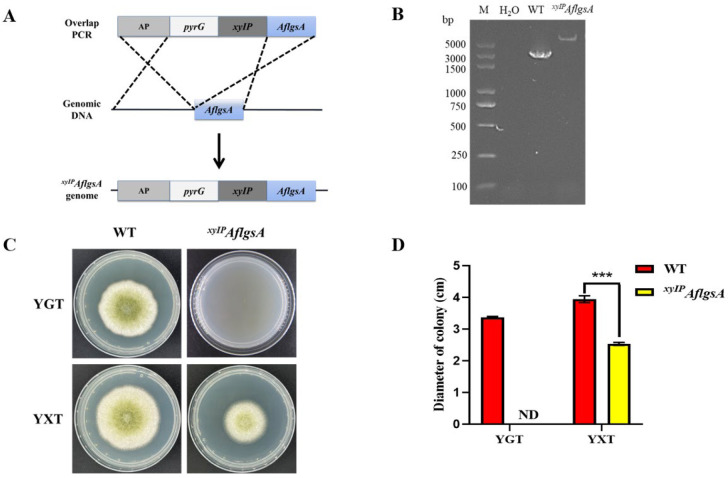
Construction and validation of the *^xyIP^AflgsA* strain of *A. flavus*. (**A**) The gene replacement strategy for the construction of *^xyIP^AflgsA*. (**B**) PCR validation of the *^xyIP^AflgsA* strain. (**C**) Phenotypic observations of the growth for WT and the *^xyIP^AflgsA* strain in YGT and YXT media in the dark at 37 °C for four days. (**D**) Statistical analysis of the colony diameters of the indicated *A. flavus* strains (panel (**C**)). ND indicates no detection. *** indicates a significance level of *p* < 0.001 based on *t*-tests with three replicates.

To investigate the function of *AflgsA* in *A. flavus*, we first tried to obtain the *AflgsA* deletion strain by homologous recombination but failed. A better alternative strategy is to construct a xylose promoter mutant strain (*^xyIP^AflgsA*) for *AflgsA* functional verification (Figure 2A). After confirmation by PCR (Figure 2B) and DNA sequencing (Figure S1), the mutant strain and WT strain were incubated in YXT medium (containing xylose) and YGT medium (without xylose) for four days at 37 °C in the dark. The mutant strain was completely unable to grow in the xylose-free medium, while growth was partially restored after the addition of xylose (Figure 2C,D). Additionally, a gradient increase in the colony diameter, mycelial tip, and conidial gemination of *^xyIP^AflgsA* strain was found with an increasing concentration of xylose (Figure S2). These results suggest that glutamine synthetase is essential for the growth of *A. flavus*.

### 2.3. Glutamine Restores the Growth Defect of the ^xyIP^AflgsA Strain

**Figure 3:**
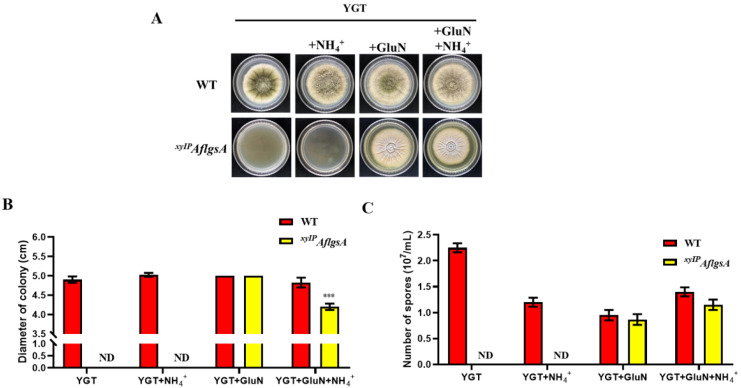
Analysis of the growth of WT and *^xyIP^AflgsA* strains. (**A**) The colony morphology of the WT and *^xyIP^AflgsA* strains on YGT medium containing 10 mM glutamine (GluN) or/and ammonium tartrate (NH_4_^+^) as nitrogen sources. (**B**) Statistical analysis of the diameter from panel (**A**). (**C**) The number of conidia produced by the above two *A. flavus* strains. ND indicates no detection. *** indicates a significance level of *p* < 0.001 based on *t*-tests with three replicates.

We found that the addition of glutamine (GluN) to the xylose-free or xylose medium restored the growth of the *^xyIP^AflgsA* strain (Figure 3A,B and Figure S3). Meanwhile, the ammonium tartrate (NH_4_^+^) or glutamate (Glu) supplement was unable to promote colony growth (Figure 3A,B and Figure S4). This suggested that the growth defect of the mutant strain was caused by the lack of glutamine synthetase. However, the *^xyIP^AflgsA* strain showed different colony color compared to the WT strain after GluN supplementation (Figure 3A). Moreover, the growth of the *^xyIP^AflgsA* strain was also inhibited by adding both GluN and NH_4_^+^ (Figure 3B,C). This phenomenon may be due to competition or inhibition of GluN uptake by excess NH_4_^+^ [21]. The mechanisms concerning the type of nitrogen sources that mediate the absorption pathway by *AflgsA* in the cell need to be further explored.

### 2.4. AflGsA Is Important for Conidia Development of A. flavus

**Figure 4:**
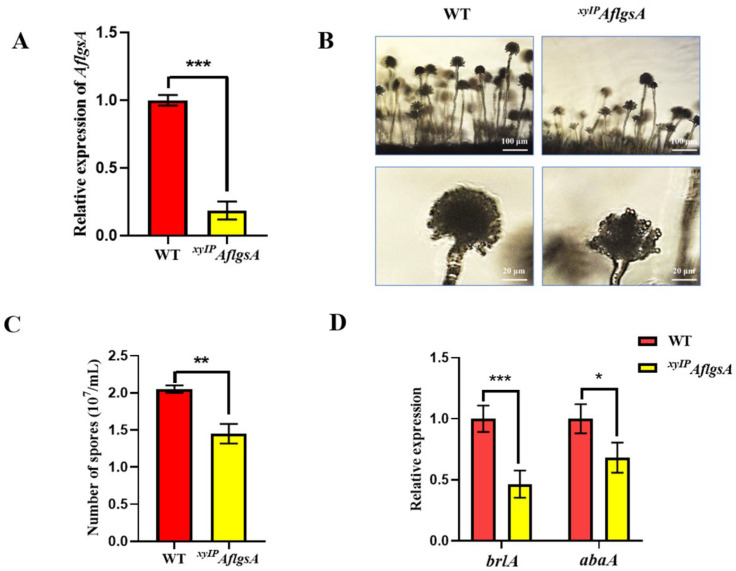
The role of AflGsA in conidia development in *A. flavus*. (**A**) qRT-PCR analysis of *AflgsA* expression in the WT and *^xyIP^AflgsA* strains in YXT medium. (**B**) Microscopic view of conidiophore formation of the above two *A. flavus* strains in YXT medium. (**C**) The number of conidia produced by the above two *A. flavus* strains in YXT medium. (**D**) Relative expression of the *brlA* and *abaA* genes in the two above strains in YXT medium with three biological replicates. * indicates a significance level of *p* < 0.05, ** indicates a significance level of *p* < 0.01, and *** indicates a significance level of *p* < 0.001 based on *t*-tests with three biological replicates. The growth conditions of the above strains are described in Section 5.4 and Section 5.6.

To further determine the effect of *AflgsA* on *A. flavus*, we monitored the transcript level of *AflgsA* in the *^xyIP^AflgsA* strain and found that it was less than half that in the WT (Figure 4A). In addition, the *^xyIP^AflgsA* strain exhibited smaller spore heads of the child seat and sparser peduncles by microscopic observation (Figure 4B). Statistical analysis also showed a significant reduction in the number of spores in the *^xyIP^AflgsA* strain compared to that in the WT strain (Figure 4C). Further analysis showed that the transcript levels of both regulatory genes for conidia, *brlA,* and *abaA*, were reduced by about half in the *^xyIP^AflgsA* strain compared to that in WT (Figure 4D). These results suggest that *AflgsA* may be involved in the upstream regulation of *abaA* and *brlA* genes during spore development of *A. flavus*, and this further regulates the production of conidia.

### 2.5. AflGsA Contributes to the Production of Sclerotia but Not to Toxin Synthesis in A. flavus

**Figure 5:**
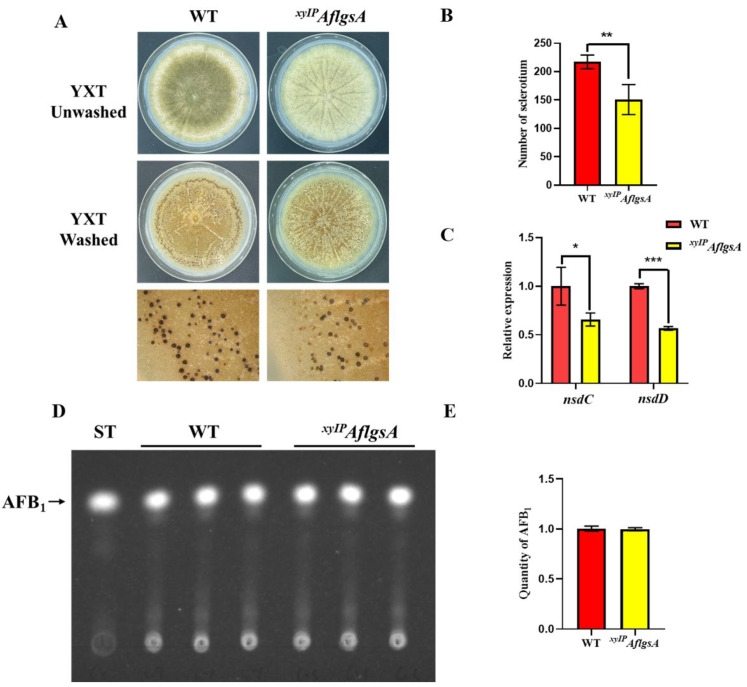
AflGsA regulates sclerotia formation in *A. flavus*. (**A**) Phenotypic observation of sclerotia formation in WT and *^xyIP^AflgsA* strains on YXT medium. (**B**) The number of sclerotia produced by the above two *A. flavus* strains. (**C**) Relative expression of the *nsdC* and *nsdD* genes in the two strains. (**D**) TLC analysis of AFB1 production in *A. flavus* WT and *^xyIP^AflgsA* strains on YXT medium containing 1 g/L MgSO_4_·7H_2_O. (**E**) Optical density analysis of AFB1 production (as in panel (**D**)). * indicates a significance level of *p* < 0.05, ** indicates a significance level of *p* < 0.01, and *** indicates a significance level of *p* < 0.001 based on *t*-tests with three biological replicates.

Sclerotia are important reproductive structures of *Aspergillus flavus*, and they aid in survival under harsh conditions. The sclerotia produced by the *^xyIP^AflgsA* strain were significantly fewer compared to those in the WT strain (Figure 5A,B). In addition, the transcript levels of the sclerotia-related genes *nsdC* and *nsdD* were significantly lower in the *^xyIP^AflgsA* strain compared to those in the WT strain, only about half of those in the WT strain (Figure 5C). In addition, the sclerotium production defect in the *^xyIP^AflgsA* strain could not be recovered by the addition of a GluN supplement (Figure S5). These results suggest that AflGsA is important for sclerotia production, and its coding gene may influence sclerotia production by being involved in the upstream regulation of *nsdC* and *nsdD*. We also investigated the production of the toxic secondary metabolite AFB1 in *A. flavus* and found no significant differences in toxin production between the *^xyIP^AflgsA* strain and the WT (Figure 5D,E). This suggests that *AflgsA* is not involved in the regulation of aflatoxin production.

### 2.6. AflGsA Balances ROS and Resists Oxidative Stress in A. flavus

**Figure 6:**
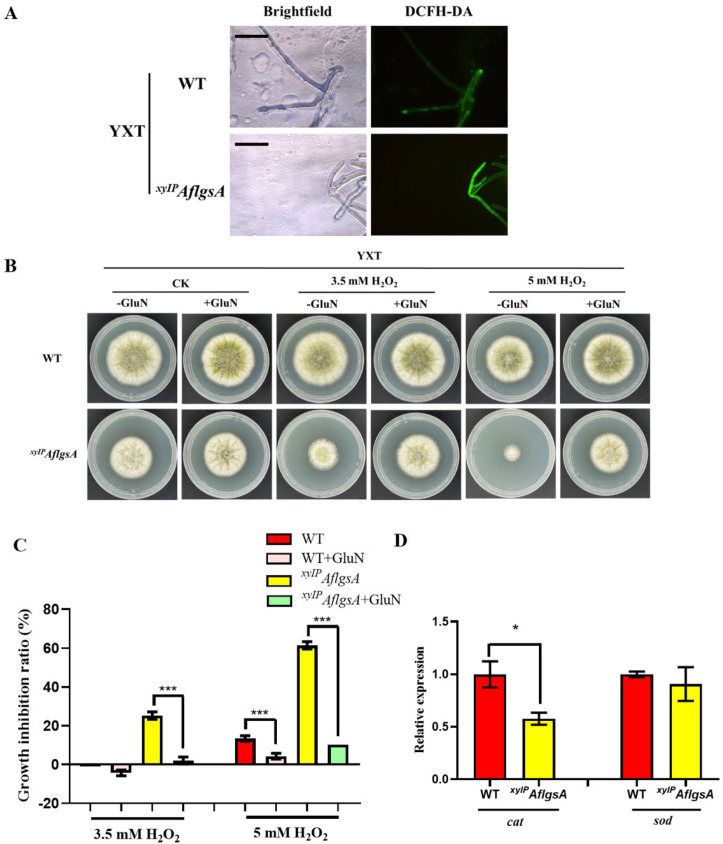
The role of AflGsA in the regulation of ROS and resistance to oxidative stress in *A. flavus*. (**A**) The ROS content of the WT and *^xyIP^AflgsA* strains was detected using fluorescence microscopy. (**B**) Growth phenotype of the WT and *^xyIP^AflgsA* strains cultured in medium (with or without GluN) with oxidative stress. (**C**) The growth inhibition rate of different strains in media under oxidative stress (as in panel (**B**)). *** indicates a significance level of *p* < 0.001 based on one-way ANOVA with three replicates. (**D**) Relative expression of the *cat* and *sod* genes in the WT and *^xyIP^AflgsA* strains. * indicates a significance level of *p* < 0.05 based on *t*-tests with three replicates. The growth conditions of the above strains are described in Section 5.4, Section 5.5, Section 5.6.

A high concentration of ROS causes oxidative damage, disrupting cell metabolism and causing apoptosis. It was found that more ROS were produced in the low-expression state of *AflgsA* (Figure 6A). When adding hydrogen peroxide to simulate the oxidative stress state, the inhibition rate of the *^xyIP^AflgsA* strain was significantly higher than that of the WT (Figure 6B,C), but this situation was significantly restored by the addition of GluN (Figure 6B,C). A similar phenotype was also shown in the addition of tBOOH to simulate the oxidative stress state (Figure S6). Further study revealed that there was no significant change in the transcript level of superoxide dismutase gene *sod*. However, the transcript level of the catalase gene *cat* was significantly lower in the *^xyIP^AflgsA* strain compared to that in the WT strain (Figure 6D). These results suggest that GluN, the catalytic product of AflGsA, plays an important role in resistance to oxidative stress.

### 2.7. AflGsA Is Involved in Light Signaling Pathways in A. flavus

**Figure 7:**
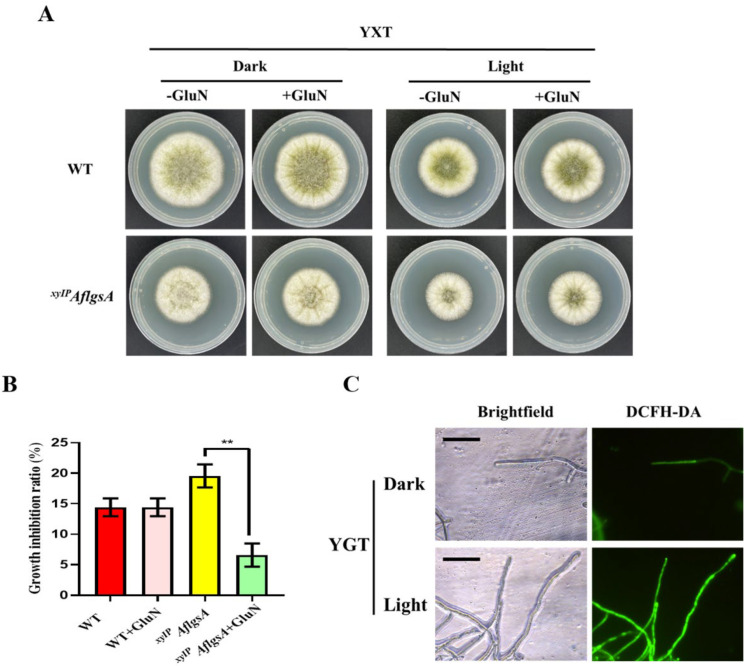
AflGsA is involved in light regulation in *A. flavus*. (**A**) Growth phenotype of the WT and *^xyIP^AflgsA* strains cultured in YXT (with GluN or without GluN) in dark or light. (**B**) Growth inhibition rate of different strains in media with light (panel (**A**)). (**C**) ROS content of the WT in dark or light. ** indicates a significance level of *p* < 0.01 based on one-way ANOVA with three replicates.

The colony diameter of *A. flavus* at 37 °C in the light was significantly smaller than that in the dark (Figure 7A). We also found that the *^xyIP^AflgsA* strain exhibited significantly higher inhibition rates under light relative to those of the WT strain (Figure 7A,B), but this inhibition of growth was restored with the addition of glutamine (Figure 7A,B). Further studies found that *A. flavus* produced more ROS under light (Figure 7C), indicating that AflGsA could regulate ROS production under light conditions.

### 2.8. Recombinant AflGsA (rAflGsA) Has Glutamine Synthetase Activity

**Figure 8:**
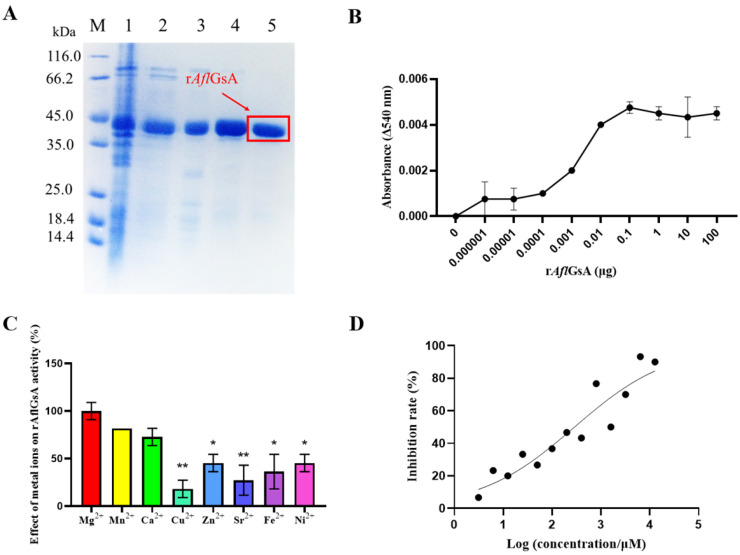
Enzymatic activity assay of recombinant AflGsA. (**A**) rAflGsA with a 6× His label was purified using Ni-NTA column chromatography. Line M: Marker; lane 1: supernatant; lane 2: wash fraction with 50 mM imidazole; lane 3: wash fraction with 100 mM imidazole; lanes 4 and 5: wash fraction with 300 mM imidazole. (**B**) Enzymatic assay of rAflGsA. (**C**) Effect of metal ions on rAflGsA activity. The activity assays were performed after incubation of the purified enzymes with 10 mM concentration of different metal chlorides for 30 min. (**D**) IC_50_ assay of L-α-aminoadipic acid on rAflGsA in vitro. * indicates a significance level of *p* < 0.05, and ** indicates a significance level of *p* < 0.01 based on one-way ANOVA with three biological replicates.

The recombinant protein rAflGsA (rAflGsA with 6× His tag, 6× His-rAflGsA) was successfully expressed and then purified by Ni-NTA column chromatography (Figure 8A). Recombinant protein activity was measured using the classical Gs enzymatic activity assay (see Section 5), and the results showed that rAflGsA has glutamine synthetase activity (Figure 8B). We further analyzed whether divalent metal ions affected the enzyme activity of rAflGsA. Based on the results of activity assays, the highest activity was observed with Mg^2+^ ions (control), while the activities with Mn^2+^ and Ca^2+^ ions were 81% and 72%, respectively (Figure 8C). Glutamine synthetase activity with the addition of Cu^2+^ ions was only 18% (Figure 8C). From the results above, it appears that the activity of rAflGsA requires the assistance of specific metal ions.

### 2.9. L-α-Aminoadipic Acid Is a Potential Inhibitor for rAflGsA and A. flavus

**Figure 9:**
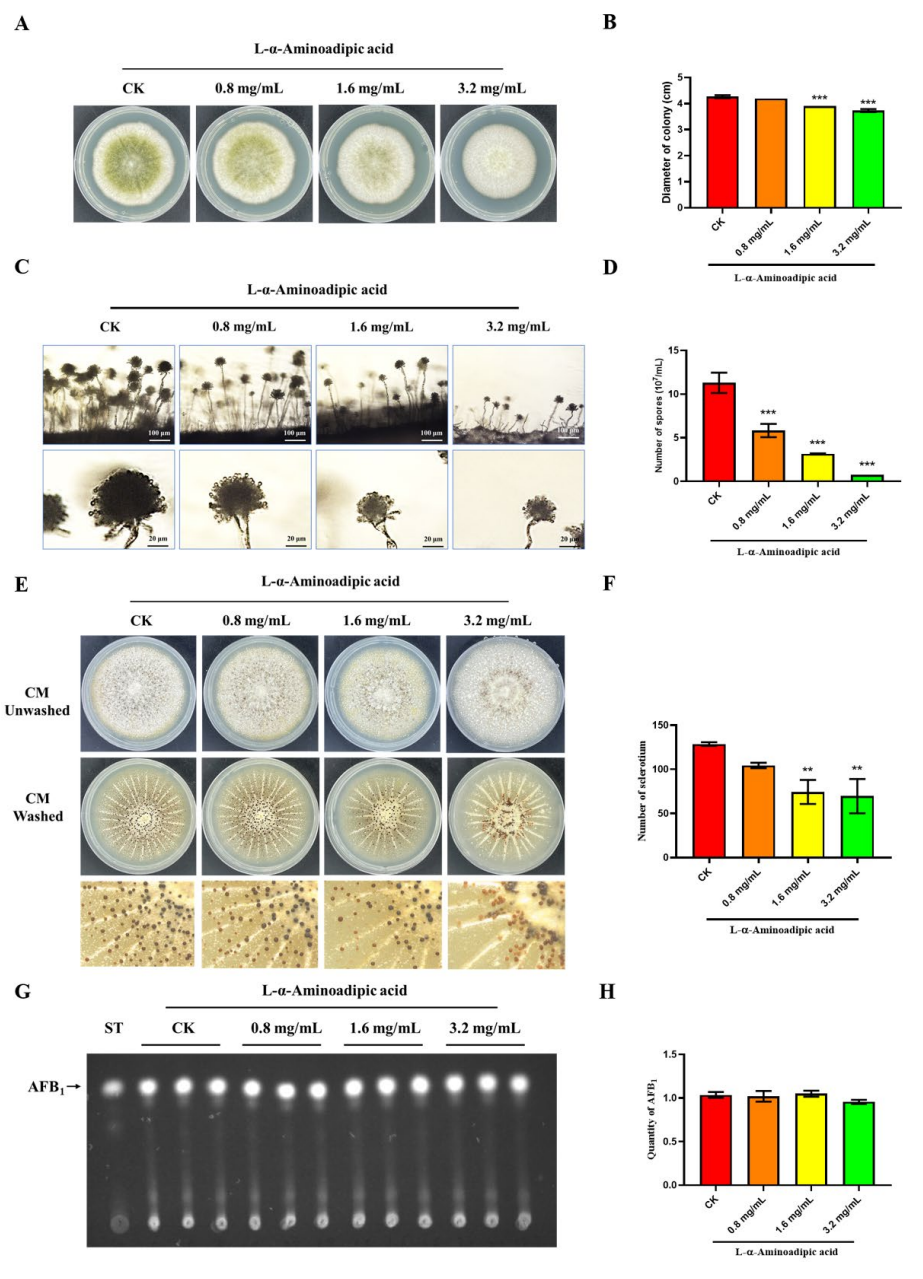
Effect of L-α-aminoadipic acid on growth, conidia production, and sclerotia formation in *A. flavus*. (**A**) The growth of the *A. flavus* WT strain was inhibited by L-α-aminoadipic acid (0–3.2 mg/mL). (**B**) Statistical analysis of the colony diameters of the WT strains treated with the inhibitor (as in panel (**A**)). (**C**) Microscopic view of conidiophore formation of the WT treated with L-α-aminoadipic acid. (**D**) The number of conidia produced by the WT strains treated with L-α-aminoadipic acid. (**E**) Phenotypic observation of sclerotia formation in the WT strains treated with different concentrations of L-α-aminoadipic acid. (**F**) The number of sclerotia produced by the WT strains (as in panel (**E**)). (**G**) TLC analysis of AFB1 production of the WT strains treated with L-α-aminoadipic acid. (**H**) Optical density analysis of AFB1 production (as in panel (**G**)). ** indicates a significance level of *p* < 0.01, and *** indicates a significance level of *p* < 0.001 based on one-way ANOVA with three biological replicates.

L-α-aminoadipic acid is a specific gliotoxin in vitro, and this chemical was reported to inhibit Gs activity in rats [31]. In this study, we found that the inhibitor L-α-aminoadipic acid suppressed the activity of rAflGsA with an IC_50_ value of 288.1 μM in vitro (Figure 8D). The results also showed that L-α-aminoadipic acid prevented the growth and conidial gemination of *A. flavus* with increasing concentrations (Figure 9A,B and Figure S7). The morphology of conidia was influenced by an increasing concentration, and the spore heads became smaller (Figure 9C). The statistics revealed a gradient decrease in the number of spores of *A. flavus* with an increasing concentration of L-α-aminoadipic acid (Figure 9D). When observing the effect of L-α-aminoadipic acid on the production of sclerotia, we found a significant decrease in the number of sclerotia when 1.6 mg/mL of L-α-aminoadipic acid was added (Figure 9E,F). In contrast, the amount of aflatoxin did not change with an increasing concentration of added L-α-aminoadipic acid (Figure 9G,H). In view of the results above, it is clear that L-α-aminoadipic acid is an effective inhibitor of rAflGsA and consequently for *A. flavus*. Therefore, we speculate that AflGsA in *A. flavus* is an ideal candidate target for the L-α-aminoadipic acid inhibitor.

## 3. Discussion

Glutamine synthetase is responsible for catalyzing the conversion of glutamine from ammonium and glutamate as well as being the central enzyme for nitrogen assimilation [32]. Unlike *Rhizobium meliloti* (*R. meliloti*) [33] and *Magnaporthe oryzae* (*M. oryzae*) [24], which have three Gs proteins, we identified only one putative glutamine synthetase (AflGsA) in *A. flavus*. Furthermore, a previous report on *R. meliloti* stated that only simultaneous knockdown of all three Gs genes could cause a complete glutamine nutritional defect [33]. In *A. flavus*, only one protein has glutamine synthetase activity, and there is no alternative pathway for glutamine synthesis. To study the biofunction of glutamine synthetase in *A. flavus*, we constructed the *^xyIP^AflgsA* mutant strain that has a complete glutamine nutrient-deficient mutation in the YGT medium.

In *A. flavus*, the addition of glutamine to the YGT medium partially restored the growth defect of the *^xyIP^AflgsA* strain. However, in contrast to the pigmented colonies with conidia produced in *A. nidulans* [21], the addition of glutamine to the *A. flavus* mutant strain resulted in the formation of pigmentation defects similar to *M. oryzae* and *G. fujikuroi* [15,24]. This suggests that the glutamine synthetase regulated the production of *A. flavus* pigments, unlike that in *A. nidulans*. In addition, the phenotype after glutamine addition was inhibited by the addition of NH_4_^+^, which may be due to the competitive inhibition of GluN by NH_4_^+^ [21]. The transcript level of *ghd* gene encoding glutamate dehydrogenase was elevated in the *^xyIP^AflgsA* strain compared to that in WT (Figure S8A). Glutamate dehydrogenase also plays an important role in ammonium assimilation. This may be a balancing mechanism for nitrogen regulation in *A. flavus*.

Like many pathogenic fungi, conidia production and sclerotia formation are important steps in the life cycle of *A. flavus*. It was found that the *AflgsA* of *A. flavus* plays an important role in the production of conidia. Further results also showed that *AflgsA* regulates spore production by regulating the transcript levels of the regulatory genes *brlA* and *abaA* for the production of conidia. Similarly to Δ*Mogln2* in *M. oryzae* [24], the inhibition of *AflgsA* function in *A. flavus* was followed by a reduction in the number of conidial peduncles and a smaller head of child seats compared to those in the WT, which may be a reason for the reduction of *A. flavus* spores. In addition, *AflgsA* in *A. flavus* regulates sclerotia formation by affecting the sclerotia-production-related genes *nsdC* and *nsdD*. All these results suggest that the sophisticated role of *AflgsA* in multiple developmental stages of *A. flavus* is related to its glutaminyl transferase activity. Glutamine synthetase in *G. fujikuroi* affects the synthesis of gibberellin (GA) and bikaverin metabolites [15]. In contrast, our study showed that both the *^xyIP^AflgsA* mutant and the inhibitor-treated strains produced aflatoxin normally, revealing that *AflgsA* is not involved in aflatoxin synthesis in *A. flavus*.

ROS is an unavoidable and harmful by-product of oxidative metabolism, and ROS dynamic balance is essential for the development of the fungus [34,35]. The inhibition of glutamine synthetase or light irradiance led to more ROS production and had an inhibitory feedback effect on growth in *A. flavus* that was restored by the addition of glutamine. Photorespiration in plants leads to high production of ROS [36], and inhibition of Gs in *A. palmeri* leads to impaired function of photorespiration accompanied by cell apoptosis [17,22]. Therefore, we hypothesized that glutamine synthetase regulated ROS-mediated inhibition of the growth of *A. flavus* under light conditions. Furthermore, glutamine synthetase can regulate the metabolism in relation to oxidative stress in cyanobacteria [37]. In *M. oryzae*, the Δ*Mogln2* strain also results in a high sensitivity to H_2_O_2_ [24]. In this study, the *AflgsA* mutant strain of *A. flavus* was highly sensitive to oxidative stress, and this sensitivity was restored by supplementing glutamine. Further studies showed that the transcript level of catalase was significantly reduced in the *AflgsA* mutant strain, suggesting that the glutamine synthetase pathway resists oxidative stress by regulating the transcriptional level of catalase.

We obtained the rAflGsA protein from *E. coli* with a purity of up to 95% and confirmed that it had glutamate synthase activity. Reports show that glutamine synthetase has positive cooperativity with different cofactors and metal ions [38,39]. Our further study clarified that the glutamine synthesis activity of rAflGsA is dependent on divalent metal ions and that the highest enzyme activity is achieved by the addition of Mg^2+^ and Mn^2+^ ions. This finding was consistent with the results of cation preference towards recombinant glutamine synthetase from *Psychrotrophic Bacterium* [40] and *Mangrove* [41].

L-α-aminoadipic acid is a specific gliotoxin in vitro [42] and is a neuroexcitatory metabolite that reduces extracellular kynurenic acid levels in a dose-dependent manner [43]. However, it is not mentioned in other studies whether L-α-aminoadipic acid has antifungal activity. Our study showed that L-α-aminoadipic acid is an inhibitor for rAflGsA in vitro, and that it also prevents the growth, spore production, and sclerotia formation of *A. flavus* in vivo. In agreement with the phenotype of the *^xyIP^AflgsA* strain, the addition of L-α-aminoadipic acid had no effect on aflatoxin synthesis. In addition, lower concentrations of L-α-aminoadipic acid (0.8 mg/mL and 1.6 mg/mL) did not affect the transcript level of *AflgsA*, but a higher dosage (3.2 mg/mL) seemed to decrease its transcriptional (Figure S8B). This suggests that L-α-aminoadipic acid may also have other unknown targets in *A. flavus*. Overall, these results suggest that L-α-aminoadipic acid is a potential inhibitor of *A. flavus*, and thus AflGsA may be an ideal target for L-α-aminoadipic acid in *A. flavus*. The study of the inhibition mechanism of L-α-aminoadipic acid against *A. flavus* is important for the prevention of *A. flavus*. This may be a very meaningful research direction in the future.

## 4. Conclusions

At present, many studies have attempted to elucidate the function of glutamine synthetase in organisms. However, the understanding of the biofunctional diversity of glutamine synthetase in filamentous fungi, especially in *A. flavus*, seems to have been relatively neglected. In this study, we found that AflGsA performed its activity as a glutamine synthetase and that it played a divergent role in the conidia production and sclerotia formation in *A. flavus*. It has an important role in the homeostasis of ROS and resistance to oxidative stress in *A. flavus*. In addition, L-α-aminoadipic acid inhibited both rAflGsA and *A. flavus* and thus was considered as a potential antifungal candidate for further study.

## 5. Materials and Methods

### 5.1. Strains and Culture Conditions

*E. coli* DH5a and BL21 (DE3) were used for plasmid DNA preparation and expression of the recombinant AflGsA (rAflGsA) protein, respectively. *A. flavus* WT and *^xyIP^AflgsA* strains were cultured in YGT (5 g/L yeast extract, 20 g/L glucose, and 1 mL/L trace elements) and YXT (5 g/L yeast extract, 20 g/L xylose, and 1 mL/L trace elements) media at 37 °C in the dark. Then, 1.5% agar was added to obtain solid media. To study aflatoxin production, YXT medium containing 1 g/L MgSO_4_·7H_2_O was used at 29 °C.

### 5.2. Sequence Analysis

The NCBI database was used to search for the *AflgsA* sequence of *A. flavus* (AFLA_051930). The homologous protein sequence of AflGsA from *A. flavus* was retrieved by BLAST. The retrieved homologous protein sequences were analyzed by domain analysis using Uniprot Tools and mapped using DOG 2.0 software. The MAGE 7.0 software was used for multiple sequence alignment of the protein sequences, and the maximum likelihood method was used to construct the phylogenetic tree.

### 5.3. Construction and Identification of Mutant Strain

All of the primers used in this study were shown in Table S1. To obtain the *^xyIP^AflgsA* mutant strain, we followed the method previously described in the literature [44]. An *AflgsA* xylose promoter mutant cassette was fused by overlapping extension PCR (*gsA-xolap-F* and *gsA-xolap-R* primers were used) to an upstream fragment of *AflgsA*, a marker gene (*A. fumigatus pyrG*), the *xyIP* xylose conditional promoter [45], and the *AflgsA* CDS fragment. *A. flavus* CA14 was used as a starting strain during the preparation of protoplasts [46]. The *AflgsA* gene’s promoter was converted to a xylose promoter by homologous recombination [44]. The *gsA-A-F* and *gsA-CDS-R* primers were used in the identification of *^xyIP^AflgsA* transformants by PCR. The *AflgsA-F* and *AflgsA-R* primers were used to identify the sequence by qRT-PCR.

### 5.4. Analysis of the Growth, Conidial Production, and Sclerotia Formation of A. flavus

For colony diameter evaluation, YGT and YXT containing 10 mM glutamine (GluN) or ammonium tartrate (NH_4_^+^) were points inoculated with 10^6^ conidia and incubated in the dark at 37 °C for four days. Spores were eluted with 2 mL of spore eluate and counted under a microscope using a hemocytometer plate to determine the number of spores after incubation in the dark at 37 °C for four days [44]. To observe conidiophore formation, 10^6^ conidia were incubated in YXT medium at 37 °C in the dark for two days, and the surface mycelium was scraped off. The colonies were cut out and placed on cover breaks and incubated for 12 h at 37 °C, then observed using a microscope. For sclerotia production analysis, 10^6^ conidia were incubated in YXT medium at 37 °C in the dark for seven days, and the morphology of sclerotia was recorded by rinsing off the mycelium with 75% ethanol [47]. For aflatoxin extraction, 10^6^ conidia were incubated in YXT containing 1 g/L MgSO_4_·7H_2_O at 29 °C in the dark for five days. The toxin in the medium was extracted using chloroform and detected using TLC [44].

### 5.5. Detection of ROS in A. flavus

To detect the ROS in *A. flavus*, WT and *^xyIP^AflgsA* strains were incubated in YXT liquid medium at 37 °C for 24 h. Mycelia were washed three times with a phosphate-buffered saline (PBS) buffer. The collected mycelia were incubated with a 10 μM DCFH-DA fluorescent probe (Beyotime, Nantong, China) for 30 min at 37 °C. After washing three times with PBS, the mycelium was placed on a slide, and ROS content was identified by microscopy. Fluorescence emission of DCFH-DA was excited at 488 nm.

### 5.6. Quantitative Real-Time PCR

The mycelia of the strains of *A. flavus* were collected after 48 h of incubation. The collected mycelia were ground in liquid nitrogen, and total RNA was extracted with the TRIzol (Biomarker Technologies, Beijing, China) reagent. RNA was translated into cDNA using a reverse transcription kit (Thermo Scientific, Waltham, MA, USA). The cDNA was then used as a template for quantitative PCR with specific primers [48].

### 5.7. Purification of Recombinant rAflGsA Protein and Determination of Enzyme Activity

The cDNA of *AflgsA* from *A. flavus* was expanded and cloned into the pET-28a expression vector. The expressed recombinant AflGsA (rAflGsA) protein was purified using Ni-NTA column chromatography [44]. The activity of rAflGsA was then measured at 540 nm using the classical glutamine synthetase enzyme activity assay [40]. Different divalent metal ions were added to assay the enzyme activity with a final concentration of 20 mM. Enzyme activity was measured after reacting at 37 °C for one hour.

### 5.8. Statistical Analysis

GraphPad Prism 5.0 (GraphPad Software, San Diego, CA, USA) was used for data statistics and analysis. All of the analyses had at least three biological replicates if not specifically indicated.

## Data Availability

Not applicable.

## Supplementary Materials

The following supporting information can be downloaded at: https://www.mdpi.com/article/10.3390/toxins14120822/s1, Table S1: Oligonucleotide primers used in this study; Figure S1: Genome sequencing validation of the *^xyIP^AflgsA* strain of *A. flavus*. *AflgsA* AP is the upstream noncoding region of the *AflgsA* gene. The mutations in the *AflgsA* CDS are synonymous mutations; Figure S2: Analysis of the growth, mycelial morphology, conidial germination, and conidiophore formation of WT and *^xyIP^AflgsA* strains. (A) The mycelial morphology of WT and *^xyIP^AflgsA* strains on the medium containing 10 g/L and 20 g/L xylose. (B) The colony morphology of WT and *^xyIP^AflgsA* strains. (C) Statistical analysis of the diameter from panel (B). (D) Conidial germination of WT and *^xyIP^AflgsA* strains. (E) Statistical analysis of the conidial germination rate from panel (D). (F) Microscopic view of the conidiophore formation of the above two *A. flavus* strains. (G) The number of conidia produced by the above two *A. flavus* strains. ND indicates no detection. ** indicates a significance level of *p* < 0.01, and *** indicates a significance level of *p* < 0.001 based on *t*-tests with three replicates. The medium containing 10 g/L xylose: 5 g/L yeast extract, 10 g/L glucose, 10 g/L xylose, 1 mL/L trace elements, and 1.5% agar. The medium containing 20 g/L xylose (YXT medium): 5 g/L yeast extract, 20 g/L xylose, 1 mL/L trace elements, and 1.5% agar; Figure S3: Analysis of the growth of WT and *^xyIP^AflgsA* strains. (A) The colony morphology of the WT and *^xyIP^AflgsA* strains on YXT medium containing 10 mM glutamine (GluN) or/and ammonium tartrate (NH_4_^+^) as nitrogen sources. (B) Statistical analysis of the diameter from panel (A). *** indicates a significance level of *p* < 0.001 based on *t*-tests with three replicates; Figure S4: Analysis of the growth of WT and *^xyIP^AflgsA* strains. (A) The colony morphology of the WT and *^xyIP^AflgsA* strains on YGT medium containing 10 mM glutamate (Glu) or on YXT medium. (B) Statistical analysis of the diameter from panel (A). ND indicates no detection. *** indicates a significance level of *p* < 0.001 based on *t*-tests with three replicates; Figure S5: AflGsA regulates sclerotia formation in *A. flavus*. (A) Phenotypic observation of sclerotia formation in WT and *^xyIP^AflgsA* strains on YXT medium containing 10 mM glutamine (GluN). (B) The number of sclerotia produced by the above two *A. flavus* strains. ND indicates no detection. * indicates a significance level of *p* < 0.05 based on *t*-tests with three replicates; Figure S6: The role of AflGsA in resistance to oxidative stress in *A. flavus*. (A) Growth phenotype of the WT and *^xyIP^AflgsA* strains cultured in medium (with or without GluN) with 0.8 mM tBOOH oxidative stress. (B) The growth inhibition rate of different strains in media under oxidative stress (as in panel (A)). *** indicates a significance level of *p* < 0.001 based on one-way ANOVA with three replicates; Figure S7: Effect of L-α-aminoadipic acid on mycelial morphology and conidial germination in *A. flavus*. (A) The mycelial morphology of the *A. flavus* WT strain was inhibited by L-α-aminoadipic acid (0–3.2 mg/mL). (B) The conidial germination of the *A. flavus* WT strain was inhibited by L-α-aminoadipic acid (0–3.2 mg/mL). (C) Statistical analysis of the conidial germination rate from panel B. ND indicates no detection. * indicates a significance level of *p* < 0.05, ** indicates a significance level of *p* < 0.01, and *** indicates a significance level of *p* < 0.001 based on one-way ANOVA with three replicates; Figure S8: Relative expression of the genes in different strains. (A) Relative expression of the *ghd* gene in the WT and *^xyIP^AflgsA* strains. (B) Relative expression of the *AflgsA* gene of the WT strains treated with the inhibitor. * indicates a significance level of *p* < 0.05 based on *t*-tests or one-way ANOVA with three replicates.
