# Supplementary material for: Glutamine Synthetase Contributes to the Regulation of Growth, Conidiation, Sclerotia Development, and Resistance to Oxidative Stress in the Fungus Aspergillus flavus
Source: Toxins (Basel). 2022 Nov 23;14(12):822. doi: 10.3390/toxins14120822 (PMC9785230; doi:10.3390/toxins14120822)
Supplement: Supplementary file 1 [file toxins-14-00822-s001.zip › toxins-2021709-supplementary.pdf]

toxins

MDPI

Article

Glutamine Synthetase Contributes to the Regulation of Growth, Conidiation, Sclerotia Development, and Resistance to Oxidative Stress in the Fungus *Aspergillus flavus*

Sen Wang, Ranxun Lin, Elisabeth Tumukunde, Wanlin Zeng, Qian Bao, Shihua Wang and Yu Wang

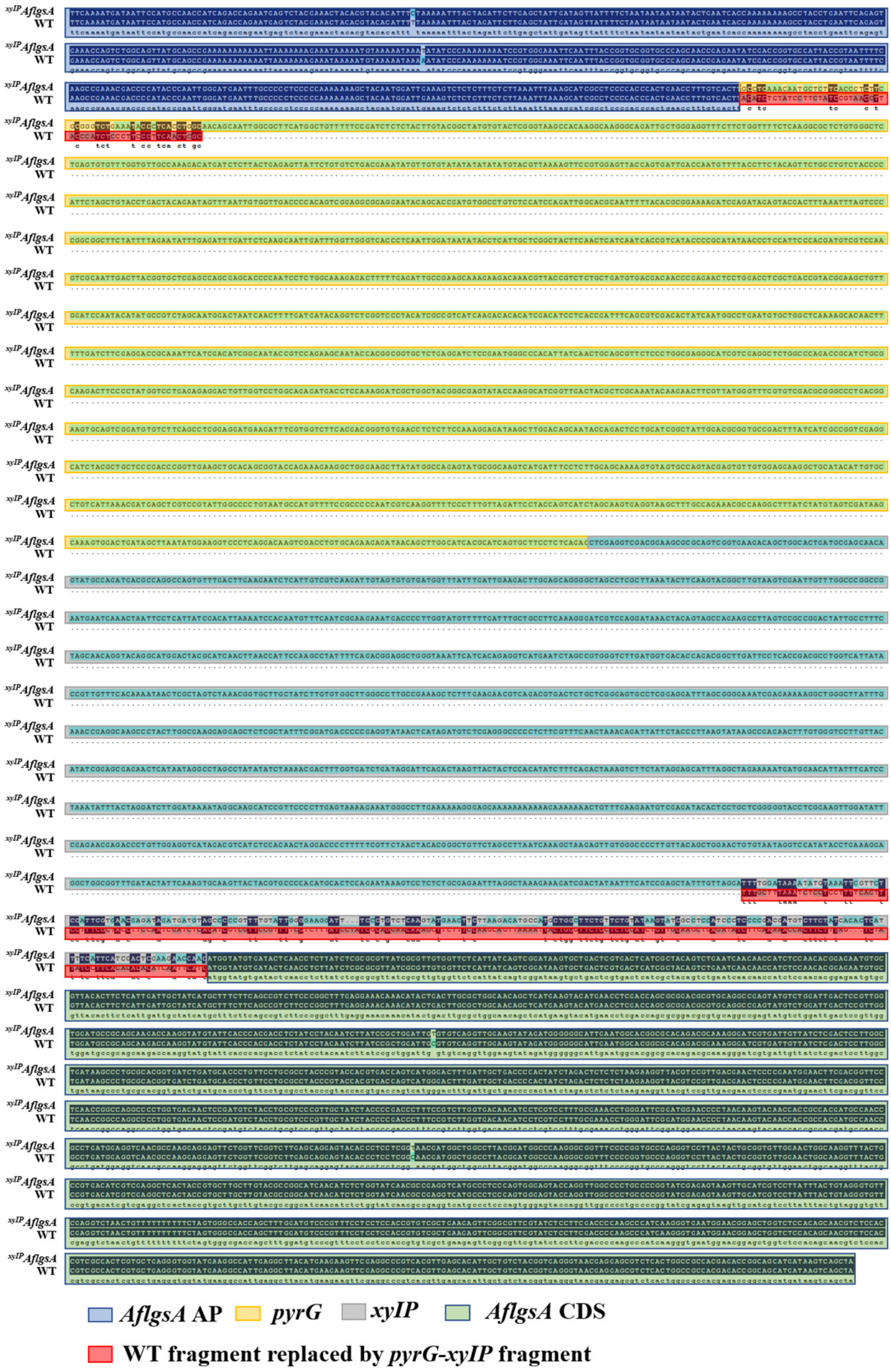

Figure S1. Genome sequencing validation of the *xyIPAflgsA* strain of *A. flavus*. *AflgsA* AP is the upstream non-coding region of the *AflgsA* gene. The mutations in the *AflgsA* CDS are synonymous mutations.

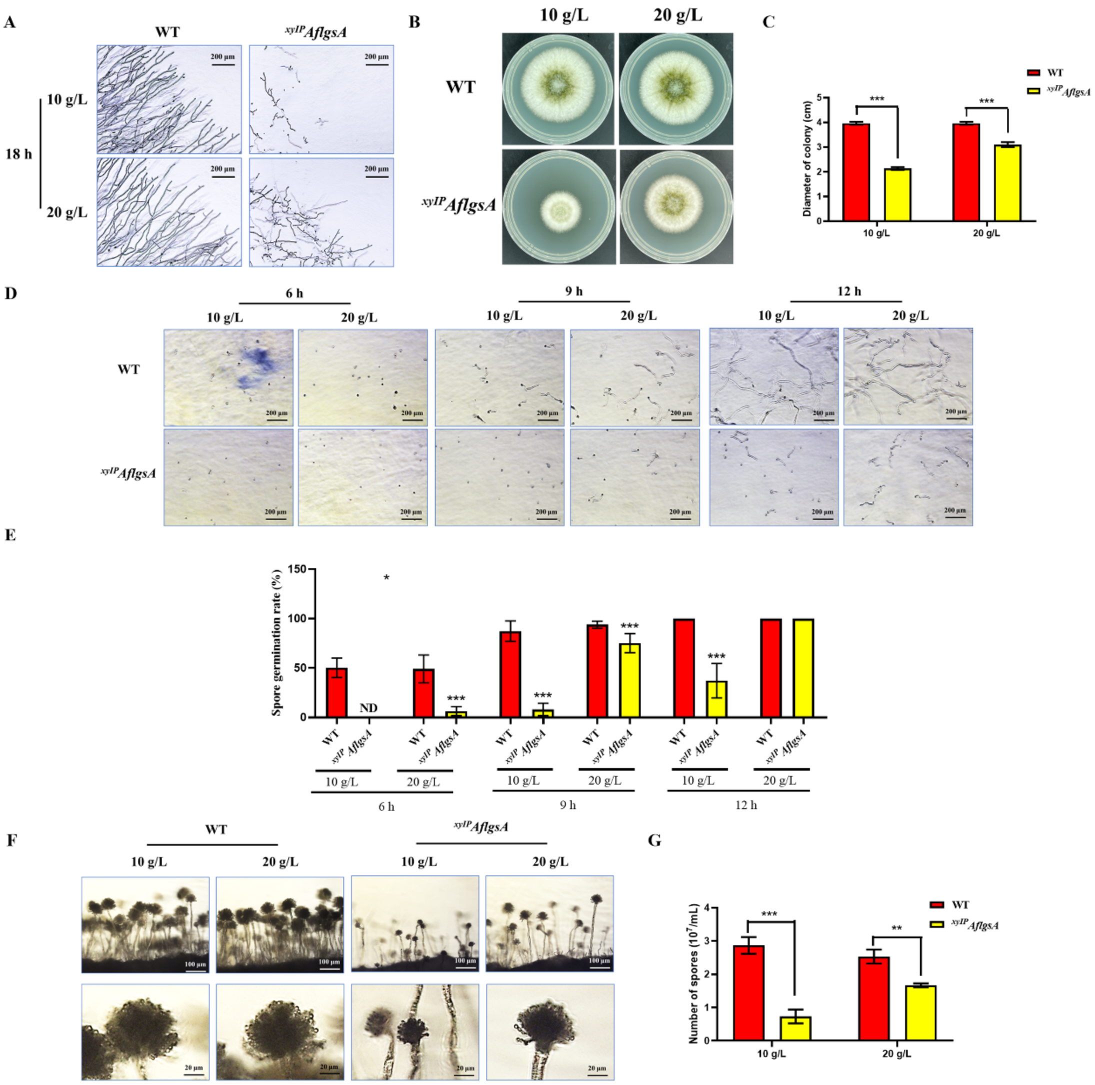

Figure S2. Analysis of the growth, mycelial morphology, conidial germination and conidiophore formation of WT and ^{xylP}*AflgsA* strains. (A) The mycelial morphology of WT and ^{xylP}*AflgsA* strains on the medium containing 10 g/L and 20g/L xylose. (B) The colony morphology of WT and ^{xylP}*AflgsA* strains. (C) Statistical analysis of the diameter from panel B. (D) The conidial germination of WT and ^{xylP}*AflgsA* strains. (E) Statistical analysis of the conidial germination rate from panel D. (F) Microscopic view of the conidiophore formation of the above two *A. flavus* strains. (G) The number of conidia produced by the above two *A. flavus* strains. ND indicates no detection. ** indicates a significance level of $P < 0.01$, and *** indicates a significance level of $P < 0.001$ based on t tests with three replicates. The medium containing 10 g/L xylose: 5 g/L yeast extract, 10 g/L glucose, 10 g/L xylose, 1 mL/L trace elements, and 1.5% agar. The medium containing 20 g/L xylose (YXT medium): 5 g/L yeast extract, 20 g/L xylose, 1 mL/L trace elements, and 1.5% agar.

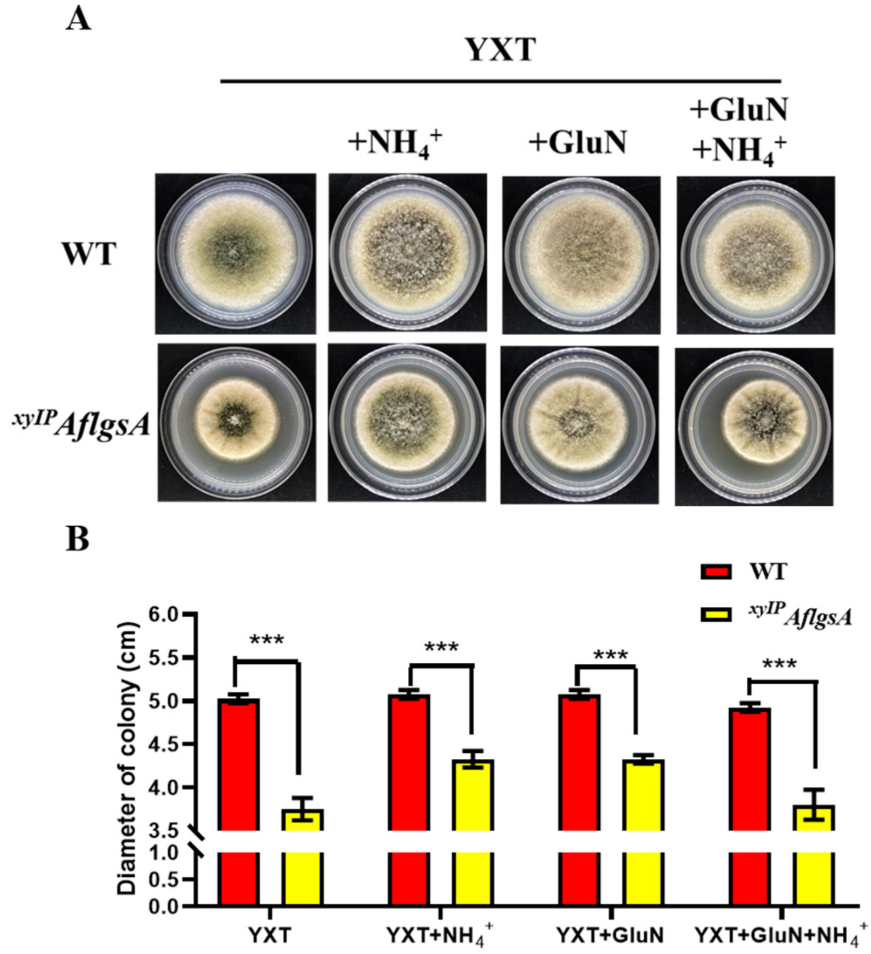

Figure S3. Analysis of the growth of WT and ^{xyIP}*AflgsA* strains. (A) The colony morphology of the WT and ^{xyIP}*AflgsA* strains on YXT medium containing 10 mM glutamine (GluN) or/and ammonium tartrate (NH_4^+) as nitrogen sources. (B) Statistical analysis of the diameter from panel A. *** indicates a significance level of $P < 0.001$ based on t tests with three replicates.

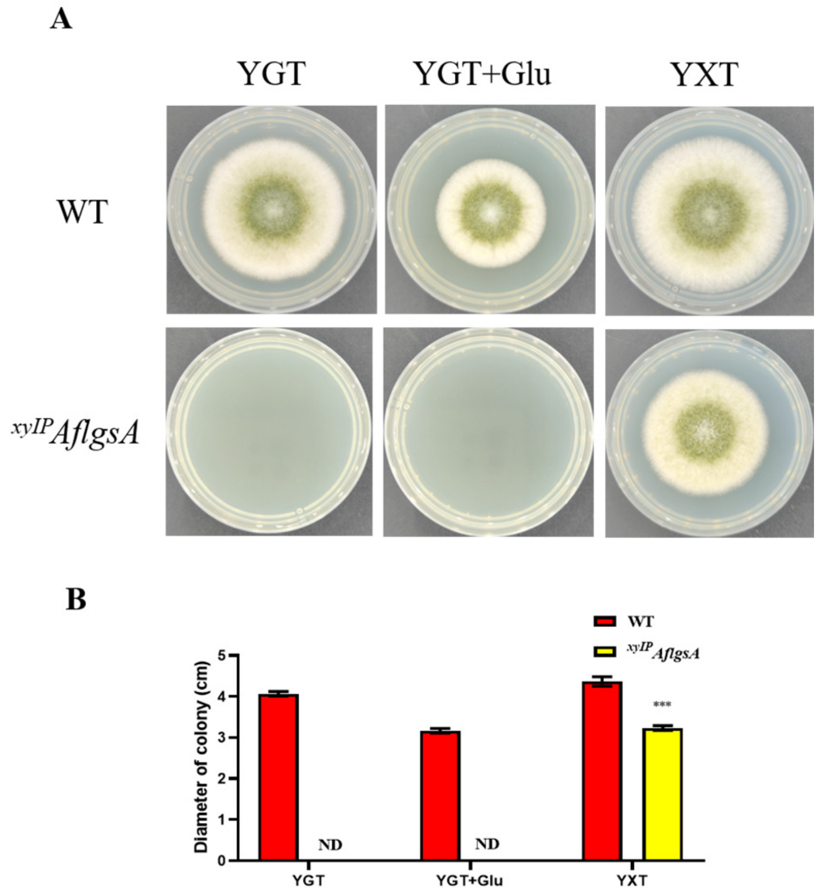

Figure S4. Analysis of the growth of WT and ^{xyIP}*AflgsA* strains. (A) The colony morphology of the WT and ^{xyIP}*AflgsA* strains on YGT medium containing 10 mM glutamate (Glu) or on YXT medium. (B) Statistical analysis of the diameter from panel A. ND indicates no detection. *** indicates a significance level of $P < 0.001$ based on t tests with three replicates.

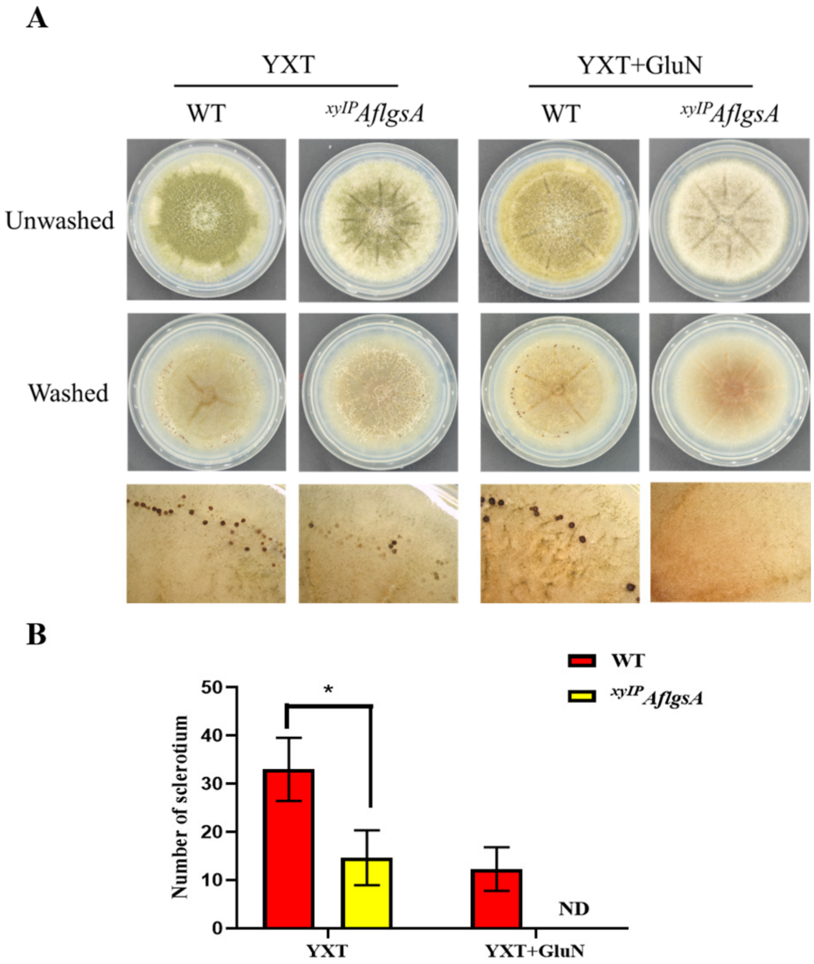

Figure S5. AflGsA regulates sclerotia formation in *A. flavus*. (A) Phenotypic observation of sclerotia formation in WT and ^{xyIP}*AflgsA* strains on YXT medium containing 10 mM glutamine (GluN). (B) The number of sclerotia produced by the above two *A. flavus* strains. ND indicates no detection. * indicates a significance level of $P < 0.05$ based on t tests with three replicates.

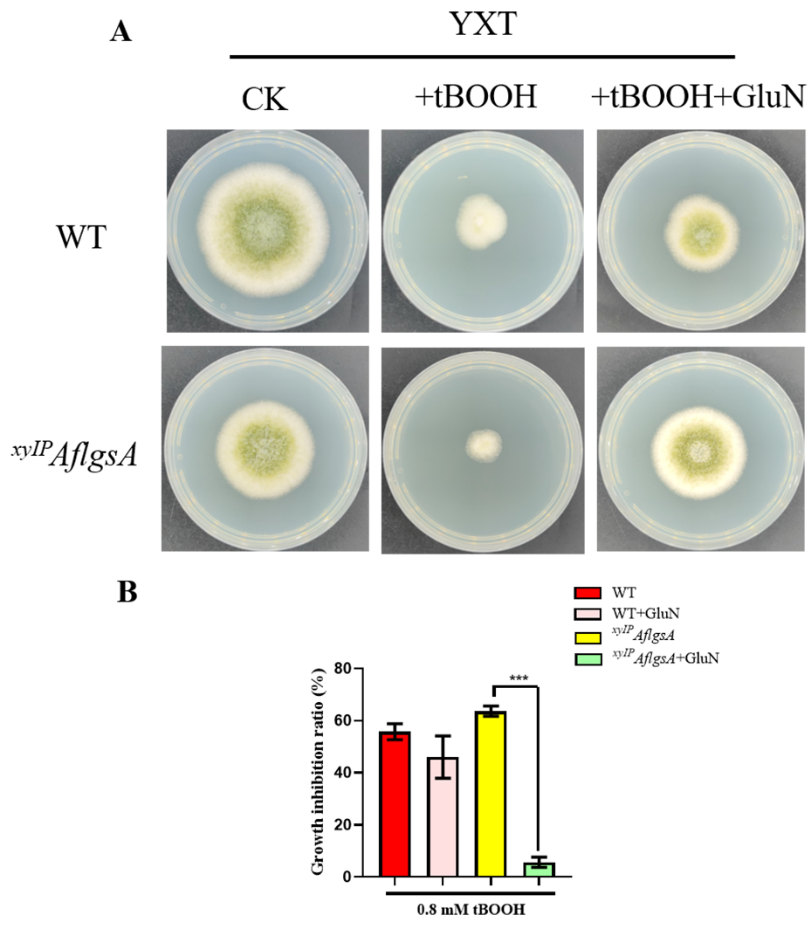

Figure S6. The role of AflGsA in resistance to oxidative stress in *A. flavus*. (A) Growth phenotype of the WT and ^{xylP}*AflgsA* strains cultured in medium (with or without GluN) with 0.8 mM tBOOH oxidative stress. (C) The growth inhibition rate of different strains in media under oxidative stress (as in panel A). *** indicates a significance level of $P < 0.001$ based on one-way ANOVA with three replicates.

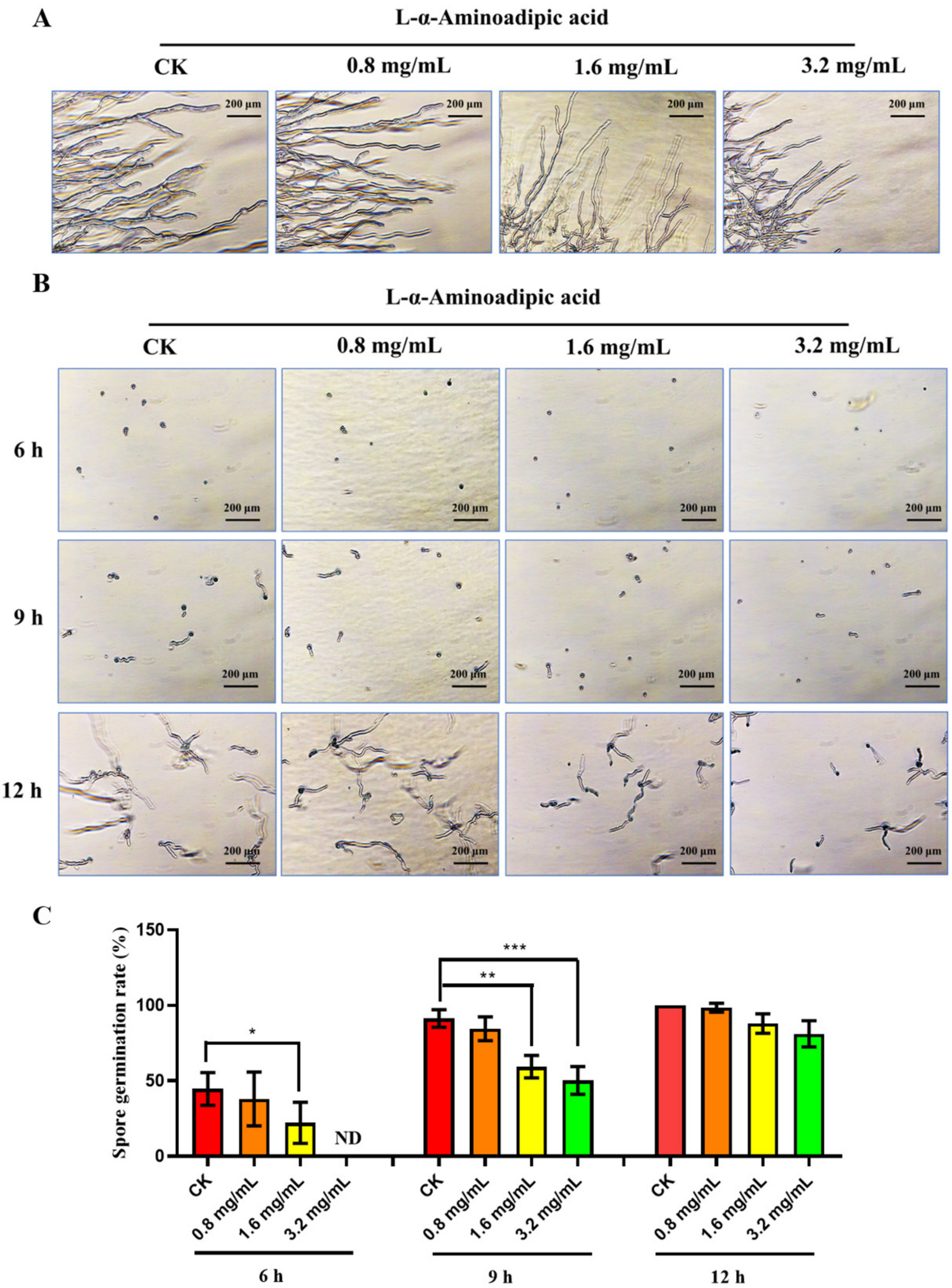

Figure S7. Effect of L-α-aminoadipic acid on mycelial morphology and conidial germination in *A. flavus*. (A) The mycelial morphology of the *A. flavus* WT strain was inhibited by L-α-aminoadipic acid (0–3.2 mg/mL). (B) The conidial germination of the *A. flavus* WT strain was inhibited by L-α-aminoadipic acid (0–3.2 mg/mL). (C) Statistical analysis of the conidial germination rate from panel B. ND indicates no detection. * indicates a significance level of $P < 0.05$, ** indicates a significance level of $P < 0.01$, and *** indicates a significance level of $P < 0.001$ based on one-way ANOVA with three replicates.

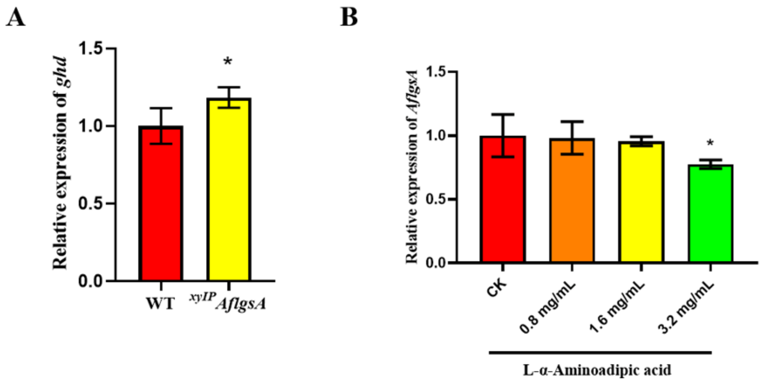

Figure S8. Relative expression of the genes in different strains. (A) Relative expression of the *ghd* gene in the WT and ^{xylP}*AflgsA* strains. (B) Relative expression of the *AflgsA* gene of the WT strains treated with the inhibitor. * indicates a significance level of $p < 0.05$ based on t tests or one-way ANOVA with three replicates.

Table S1. Oligonucleotide primers used in this study.

Primers	Sequence (5′-3′)	Application	
gsA-A-F	GGGGTGCGATTATCTGGG	To amplify *AflgsA* upstream fragment	Construction of ^{xylP}*AflgsA* transformants
gsA-A-R	GGGTGAAGAGCATTGTTTGAGGCAAGTGACAAAGGTTCAGTGGG		
pyrg-xylP-F	GCCTCAAACAATGCTCTTCACCC	To amplif of *pyrG* and *xylP* fusion fragment	
pyrg-xylP-R	GTTGGTTCTTCGAGTCGATGAATG		
gsA-CDS-F	CATTCATCGACTCGAAGAACCAACATGGTATGTGATACTCAACCTCT	To amplify *AflgsA* CDS fragment	
gsA-CDS-R	TGAACTTATCAATGCTGCC		
gsA-xolap-F	TCTATCGCATCGGACTGGC	Nest-primers for fusion PCR	
gsA-xolap-R	GAGACGCTCCTCGTTACCCT		
gsA-A-F	GGGGTGCGATTATCTGGG	Identification of ^{xylP}*AflgsA* transformants by PCR	
gsA-CDS-R	TGAACTTATCAATGCTGCC		
gsA-GS-F	TGAAACACCGCAACAACG	Identification of ^{xylP}*AflgsA* transformants by Genome sequencing	
gsA-GS-R	GGAATACGAATGCTACCACCAC		
AflgsA-F	TGTCTACCTGCGTCCCGTTGC	qRT-PCR primers to detect the *AflgsA* gene transcript level	for cDNA-qRT-PCR
AflgsA-R	CGTGGCGGTGGTTGTACTTGTT		
brlA-F	GCCTCCAGCGTCAACCTTC	qRT-PCR primers to detect the *brlA* gene transcript level	
brlA-R	TCTCTTCAAATGCTCTTGCCTC		
abaA-F	CACGGAAATCGCCAAAGAC	qRT-PCR primers to detect the *abaA* gene transcript level	
abaA-R	CATCACAATCCCTTACCTCCC		
nsdD-F	GGACTTGCGGGTCGTGCTA	qRT-PCR primers to detect the *nsdD* gene transcript level	
nsdD-R	AGAACGCTGGGTCTGGTGC		
nsdC-F	GCCAGACTTGCCAATCAC	qRT-PCR primers to detect the *nsdC* gene transcript level	
nsdC-R	CATCCACCTTGCCCTTTA		
ghd-F	TCCGAGGCTTCCATCTACG	RT-PCR primers to detect the *ghd* gene transcript level	
ghd-R	GAACAGTGAGCGGGCATTA		
cat-F	TTTCTTCACGGCTCCTGGAC	RT-PCR primers to detect the *cat* gene transcript level	
cat-R	CGAGCTCTTGACGTTGGAGT		
sod-F	CATGTCCACCAGTTCGGTGA	RT-PCR primers to detect the *sod* gene transcript level	
sod-R	GCATCGGTCTTGAAGTTGCC		
β-actin-F	ACGGTGTCGTCACAAACTGG	qRT-PCR primers to detect the *β-actin* gene transcript level	
β-actin-R	CGGTTGGACTTAGGGTTGATAG		
β-tubulin-F	CAACAACTCCAGACCGCC	qRT-PCR primers to detect the *β- tubulin* gene transcript level	
β-tubulin-R	CTCGTCCATACCCTCACC		
